# A Multi-Chamber Paper-Based Platform for the Detection of Amyloid β Oligomers 42 via Copper-Enhanced Gold Immunoblotting

**DOI:** 10.3390/biom11070948

**Published:** 2021-06-26

**Authors:** Le-Minh-Tu Phan, Sungbo Cho

**Affiliations:** 1 School of Medicine and Pharmacy, The University of Danang, Danang 550000, Vietnam; 2 Department of Electronic Engineering, Gachon University, Seongnam 13120, Gyeonggi-do, Korea; 3 Department of Health Sciences and Technology, GAIHST, Gachon University, Incheon 21999, Korea

**Keywords:** Alzheimer’s disease, amyloid beta 42 oligomers, multi-chamber, wax printing, copper-enhanced gold nanoprobe, colorimetric immunoblot

## Abstract

The early diagnosis of Alzheimer’s disease (AD) remains a challenge for medical scientists worldwide, leading to a number of research efforts that focus on biosensor development for AD biomarkers. However, the application of these complicated biosensors is limited in medical diagnosis, due to the difficulties in robust sensing platform development, high costs, and the necessity for technical professionals. We successfully developed a robust straightforward manufacturing process for the fabrication of multi-chamber paper devices using the wax printing method and exploited it to detect amyloid beta 42 oligomers (AβO42, a significant biomarker of AD) using copper-enhanced gold nanoprobe colorimetric immunoblotting. Small hydrophilic reaction chambers could concentrate the target sample to the desired size to improve the sensing performance. The copper-enhanced gold nanoprobe immunoblot using the designed multi-chamber platform exhibited a highly sensitive performance with a limit of detection of 320 pg/mL by the naked eye and 23.7 pg/mL by a smartphone camera. This process from sensing manufacture to sensing conduction is simple to perform whenever medical technicians require time- and cost-savings, without complicated instruments or the need for technical professionals, making it feasible to serve as a diagnostic tool worldwide for the early monitoring of AD and scalable devices for the sensing application of various biomarkers in clinical settings.

## 1. Introduction

The development of an accurate diagnostic tool for Alzheimer’s disease (AD), the most common cause of dementia worldwide, has attracted widespread scientific interest. Amyloid beta (Aβ) and tau protein with significant effects in AD pathogenesis are considered as core biomarkers of AD [1]. Among the various hypotheses for AD progression, the amyloid hypothesis has been a long-term adherent to the AD in the impairment of neuronal and cognitive functions, due to the formation of plaque in the brain by the accumulation of Aβ fragments and aggregates [2,3]. There are two main isoforms of the Aβ species (Aβ40 and Aβ42) that are the most abundant that appear in the brain [4]. Although Aβ40 is more abundant than Aβ42 in the brain [5,6], Aβ40 is present only in a subset of amyloid plaques, while Aβ42 is the main Aβ species in amyloid plaques, suggesting that the aggregation of Aβ42 may precede that of Aβ40 [4,7]. Although the structural difference between Aβ40 and Aβ42 is only two amino acid residues, Aβ42 exhibits a higher neurotoxicity than Aβ40 [8,9]. Aβ42 oligomers (AβO42) play a critical role in neuronal death and cognitive dysfunction, which inhibits neuronal viability more than 10-fold compared to fibrils and more than 40-fold compared to peptide, with induced inhibition significant at 10 nM [10,11,12,13]. Hence, due to the significant inhibitory effect of low concentrations of AβO42, the early detection of AβO42 is essential for the development of a highly sensitive biosensor, contributing to the efficient intervention of AD.

To develop sensitive biosensors, many potential optical and electrochemical sensors have been fabricated to monitor AβO [14]. Although these methods, such as magnetic resonance imaging, surface plasmon resonance, and surface-enhanced Raman scattering exhibit highly sensitive efficacy, they are not particularly applicable in commercial medical application, due to their requirements which include complicated instruments, and costly and time-consuming processes. To address these issues, a sensitive biosensor for AβO should be developed with advantages that include low-cost operation, portability, and ease-of-use. Paper-based analytical devices provide a leading alternative point-of-care test due to their portability, ease of fabrication, and equipment independence [15,16]. The wax printing method for the fabrication of paper-based devices requires only a commercial printer and a heating source, making it suitable for high throughput production in medical diagnostic applications [17]. Therefore, wax printing-supported paper-based devices could play an important role as alternative devices for the monitoring of AβO, exhibiting applicable potential as a medical diagnostic portable device.

**Figure 1:**
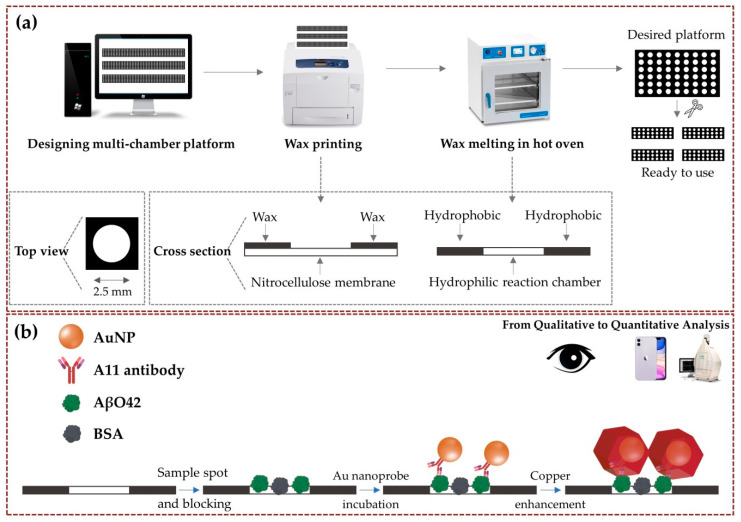
Schematic from the fabrication of the multi-chamber paper-based platform to the detection of AβO42. (**a**) Straightforward formation process of the multi-chamber platform using the wax printing method. After printing wax toner on NCM, wax penetrates the membrane to form a hydrophobic pattern by heating in a hot oven, forming the hydrophilic reaction chamber that concentrates the sample in the designed size. (**b**) Colorimetric monitoring of AβO42 concentration via copper-enhanced Au nanoprobe immunoblotting.

In this study, we fabricated a multi-chamber paper-based platform using the wax printing method for the sensitive detection of AβO42 via copper-enhanced gold nanoparticles (AuNPs)-based immunoblotting (Figure 1). Multi-chambers on a nitrocellulose membrane (NCM) were designed in a square array of circular shapes of 2.5 mm diameter for each chamber, with a 2.5 mm distance between them. A hydrophilic reaction chamber was formed after the formation of hydrophobic patterns by heating wax printed NCM. The small size of each reaction chamber supported the concentration of the amount of AβO42 antigen in the hydrophilic region. The specific AβO42 antibody conjugated AuNPs were then treated on the multi-chamber platform, followed by the colorimetric signal amplification using copper-enhanced methods [18,19]. The complex between copper ion Cu^2+^ and polyethyleneimine was reduced by sodium ascorbate (SA) to grow on the surface of AuNPs, leading to signal amplification. The target AβO42 can be detected by the color intensity of each reaction chamber that corresponded to the concentration of AβO42 with a wide range of concentrations. The manufacturing of multi-chamber paper-based platforms exhibits promising potential for the monitoring of AβO42 even in developed and poor countries, due to their advantages that include simplicity, a low-cost operation, instrument independence, and a highly sensitive performance.

## 2. Materials and Methods

### 2.1. Materials

A nitrocellulose membrane (Nupore, Ghaziabad, India) was used to fabricate the multi-chamber paper devices. A wax printer (ColorQube 8570, Xerox, Seoul, Korea) was utilized to print hydrophobic patterns on the substrates of NCM. Tetrachloroauric(III) acid (HAuCl_4_·3H_2_O) (Sigma–Aldrich, Seoul, Korea) and sodium citrate dehydrate (C_6_H_5_Na_3_O_7_·2H_2_O) (OCI, Seoul, Korea) were used to synthesize gold nanoparticles. The copper-enhancing solution was freshly made from branched polyethylenimine (BPEI, average molecular weight 1800), copper chloride dihydrate (CuCl_2_·2H_2_O), and sodium ascorbate, purchased from Alfa Aesar, Seoul, Korea. Poly(ethylene glycol) (PEG, average molecular weight 200) was obtained from Sigma–Aldrich, Seoul, Korea. Lyophylized AggreSure^™^ β-amyloid (1-42) peptide (Anaspec, CA, USA) was used as target AβO42, in accordance with the literature [20,21] that confirmed the highly concentrated AβO42 in this product. Aβ42 monomer, phosphorylated tau protein (p-tau231), C-reactive protein (CRP), and tumor necrosis factor α (TNF-α) were obtained from Alfa Aesar, Seoul, Korea. Aligomer A11 polyclonal antibody (Invitrogen, Seoul, Korea) acted as a specific AβO42 antibody to recognize the AβO42 target. Deionized water at 18.2 MΩ cm was purified using a water purification system (Purescience, Porirua, New Zealand). All reagents from commercial sources were of analytical grade and were used directly, without further purification.

### 2.2. Synthesis of AuNP and Conjugation of A11 Antibody

Spherical AuNPs were successfully synthesized using a citrate reduction of HAuCl_4_ based on the Turkevich method [22]. Briefly, a HAuCl_4_ solution (100 mL, 1 mM) was freshly prepared under vigorous stirring. Sodium citrate was then quickly injected into gold aqueous solution, after which the solution was heated to 95 °C on a hotplate with constant stirring. The reduction reaction continuously occurred at a constant temperature until the solution color turned to dark red, indicating the successful formation of monodispersed AuNPs. The reaction solution was then cooled naturally to room temperature, and stored in the dark at 4 °C for further use.

For the conjugation of the antibody to the AuNPs, PEG200 was first added to the concentrated solution of AuNPs (1 mL, pH 8.5) to increase the stability of monodispersed AuNPs, followed by the injection of the specific anti-AβO42 antibody A11 (50 µL, 100 µg/mL). Afterwards, the solution was gently mixed and incubated at 4 °C for 2 h to accelerate the immobilization of the antibody onto the surface of the AuNPs by electrostatic attraction [23]. Purified AuNPs@A11 nanoprobe solution was obtained and resuspended in a 5 mL buffer (tris-buffered saline with 0.1% Tween 20), after washing by centrifugation (4 °C, 12,000 rpm, 30 min). The Au nanoprobes were then stored at 4 °C for further use. Au nanoprobes were inoculated into the copper-enhancing solution to grow into polygonal core–shell Au–Cu nanocomposites.

UV–visible spectra were recorded by Epoch2 Microplate Spectrophotometry (Biotek, Korea). TEM imagery of AuNPs was obtained by high-resolution transmission electron microscopy (FEI Tecnai, Oregon, USA) at a 300 kV voltage. SEM imagery of the polygonal core-shell Au–Cu nanocomposites was measured by scanning electron microscopy FE-SEM (Carl Zeiss, UK).

### 2.3. Fabrication of Multi-Chamber Paper-Based Platform Using Wax Printing Method

Figure 1a describes in detail the step-by-step fabrication of the multi-chamber using wax printing. The designed multiplex paper-based platform was made using Microsoft Powerpoint software (Microsoft office 365) with a square array of circular chamber sizes of 2.5 mm and a distance between the chambers of 2.5 mm. The hydrophobic pattern was printed on NCM using a wax printer with maximum resolution. The printed NCM was heated in an oven at 95 °C for 2 min to melt the wax toner and penetrate into NCM, then cut into the desired number of chambers, which were then ready to use, or were stored at an ambient condition for further use. Coomassie brilliant blue dye was used to confirm the successful hydrophobic pattern onto NCM after heating.

### 2.4. Copper-Enhanced Gold Immunoblotting for Quantitative Measurement of AβO42

Figure 1b clearly illustrates the copper-enhanced gold immunoblotting for the detection of the AβO42 target. Briefly, multiplex paper-based reaction chambers were pretreated with a TBST buffer for 5 min and dried at 37 °C. AβO42 (1 µL) at different concentrations of (0–1) µg/mL was dropped onto the reaction chambers, and dried at 37 °C for 10 min, repeating two times. A BSA solution (3% *w*/*v*) was then used to block the reaction chambers for 30 min, by preventing non-specific absorption on the chambers. After washing the multiplex paper-based platform with a TBST buffer 2 times, this device was then incubated with the A11 antibody conjugated AuNPs solution for 1 h under gentle shaking. The multiplex paper-based devices were immersed in fresh copper-enhancing solution (CuCl_2_ (1 mL, 0.1 M), BPEI1800 (1 mL, 0.5% *w*/*v*), SA (10 mL, 0.1 M)) for 10 min. A smartphone camera and a ChemiDoc MP Imaging System (Bio-Rad, Seoul, Korea) were used to photograph the multi-chamber devices, and the color intensity of each chamber was analyzed by ImageJ software with the quantification relative to the background of NCM, which corresponded to the concentration of AβO42.

## 3. Results

### 3.1. Formation Process of the Polygonal Core-Shell Au–Cu Nanocomposite

**Figure 2:**
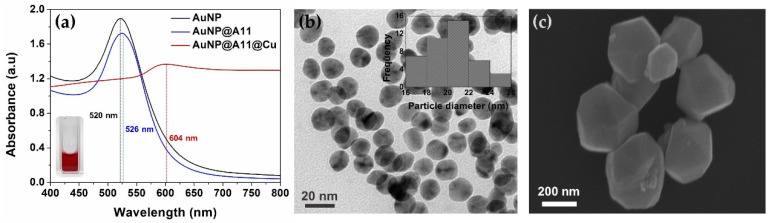
Illustration of the proposed progress from AuNP synthesis to the conjugation of the antibody onto AuNP and the modification of the conjugated AuNPs with copper; (**a**) Absorption spectra of AuNP, A11 conjugated AuNP, and copper-enhanced gold AuNPs@A11@Cu. The absorption peaks shifted from 520 nm for AuNP (inset is the photo of AuNPs solution) to 526 nm after antibody conjugation, then 604 nm after size enhancement by copper; (**b**) TEM image of monodispersed AuNPs, inset is the size distribution of AuNP by dynamic light scattering; (**c**) SEM image of polygonal core–shell Au–Cu nanocomposites.

The successful formation of the polygonal core–shell Au–Cu nanocomposite after the inoculating antibody conjugated the AuNPs into a copper reduction solution could support the possibility of colorimetric signal amplification using copper-enhanced gold immunoblotting on the multi-chamber platform. The relatively monodispersed AuNPs were successfully synthesized with a hydrodynamic size of 20.4 ± 2.2 nm (inset of Figure 2b), slightly higher than the diameter from the TEM image (around 17 nm) due to the interference of the dispersant (Figure 2b). Figure 2a shows the deep red color of the AuNPs solution under daylight (inset of Figure 2a), and the absorption spectra of the step-by-step formation of the polygonal core–shell Au–Cu. The absorption peak shift from 520 nm of AuNPs to 526 nm of A11 antibody conjugated AuNPs (AuNP@A11) confirmed the successful immobilization of the antibody onto the surface of AuNP [24,25]. The conjugated AuNP@A11 was inoculated into the copper-enhancing solution for 10 min, resulting in the formation of the polygonal core–shell Au–Cu nanocomposites with an enlarged size and shape change (Figure 2c), leading to the possibility of this process supporting copper-enhanced gold immunoblotting on the multi-chamber platform.

### 3.2. Confirmation of the Successful Fabrication of Wax Printed Multi-Chamber Paper

**Figure 3:**
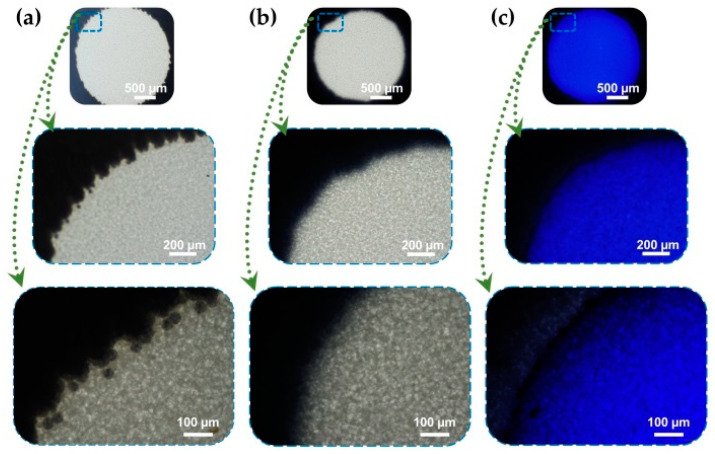
Paper-based reaction chambers by wax printed on NCM under microscopic observation at different magnifications. Scale bars are (500, 200, and 100) µm, respectively. (**a**) Surface of wax printed chamber before heating that shows the presence of spherical toner particles; (**b**) Wax particles melt and penetrate into NCM to form hydrophobic patterns upon 90 °C heating for 2 min; (**c**) Hydrophilic reaction chamber is separated with hydrophobic patterns with confirmation from using Coomassie brilliant blue dye.

The successful fabrication of the wax printed multi-reaction chambers on NCM was confirmed using blue dye under microscopic photography observation. Figure 3 shows the photographs of the as-prepared reaction chamber before and after heating. Blue dye was used to verify the ability of the chamber to concentrate the solution by the separating capacity of the hydrophobic patterns. Small wax toner particles were observed after printing wax onto NCM (Figure 3a), which were then melted, and all the wax penetrated inside the NCM under a heating effect to form the hydrophobic patterns (Figure 3b). Blue dye solution was dropped onto the hydrophilic reaction chamber, and did not spread toward the hydrophobic barriers, suggesting the effective centralization of the as-prepared multi chambers.

### 3.3. Feasibility of Using Copper-Enhanced Gold Immunoblotting to Measure AβO42

**Figure 4:**
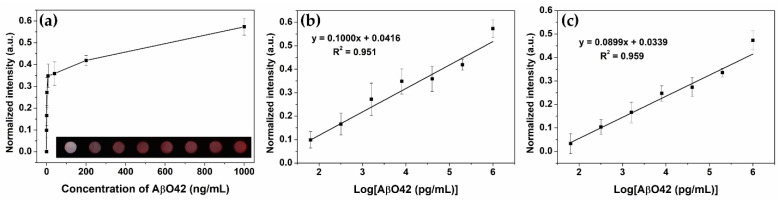
Quantitative monitoring of AβO42 by the copper-enhancing Au nanoprobe immunoblotting using the multi-chamber paper-based platform. (**a**) Normalized color intensity of the reaction chambers with different concentrations of AβO42 of (0–1) µg/mL). Inset is the photograph of the multi-chamber devices using a smartphone camera after conducting immunoblotting at increasing concentrations. (**b**) AβO42 calibration curve plotted on a logarithm scale using a smartphone camera. (**c**) AβO42 calibration curve plotted on a logarithm scale using the ChemiDoc Imaging System.

After preparing the multi-chamber platforms, they were utilized to monitor the concentration of the AβO42 target by a copper-enhanced gold immunoblot. After the immune process, a smartphone camera and the Chemidoc system were used to capture the color of the finalized platform (inset of Figure 4a), suggesting the ability of naked-eye visualization as low as 320 pg/mL, and were then used to calculate the color intensity of each chamber (Figure 4a). The normalized color intensities of the chambers were increased by increasing the concentration of AβO42 of (0–1) µg/mL), showing good linear relationships: R^2^ = 0.951 with a smartphone camera (Figure 4b) and R^2^ = 0.959 with the Chemidoc image system (Figure 4c). The limit of detection was calculated as 23.7 and 17.5 pg/mL for the smartphone camera and the Chemidoc system, respectively, indicating the feasibility of the copper-enhanced gold immunoblot using the as-prepared multi-chamber paper-based platform.

**Figure 5:**
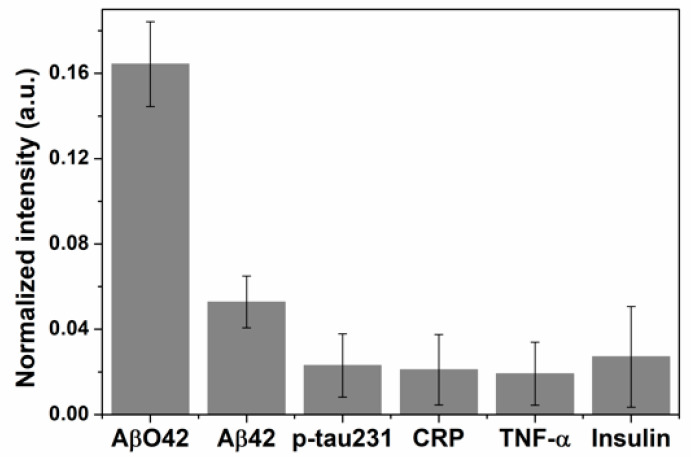
Specificity of the copper-enhanced god immunoblotting for AβO42 target compared to interferences that include Aβ42 monomer, p-tau231, CRP, TNF-α, and insulin.

The specificity of this immunoblot technique was verified by applying the method mentioned above with different human biomarkers that included Aβ42 peptide, phosphorylated tau protein (p-tau231), C-reactive protein (CRP), tumor necrosis factor α (TNF-α), and insulin at a double concentration of AβO42 (Figure 5). These interferences did not display any significant color intensity, whereas the color intensity of the AβO42 reaction chamber was remarkably higher, indicating the high specificity toward AβO42 of this immunoblotting approach compared to several measured biomarkers.

## 4. Discussion

Alzheimer’s disease, a common disease in the elderly, has continued to progress in both developed and underdeveloped countries, leading to a huge economic and social burden worldwide. Therefore, it is essential to develop a simple and affordable platform for the accurate diagnosis of AD. With the combination of tau protein as a core biomarker for the diagnosis of AD, AβO42 is considered as a significant marker for the early monitoring of AD [26]. However, the conventional diagnostic tools or the as-developed biosensors for biosensing diagnostics face limits which include unavailability, difficulties in the sensor fabrication process, an inability to allow scalable and robust fabrication, cost- and time-consuming features, and the requirement for complex instruments and technical professionals. Hence, we have developed an applicable process from the straightforward manufacture of a sensing platform to the finalization of results.

Wax printing is considered a promising technique for the fabrication of a paper-based platform, due to its simplicity, cost-effectiveness, and suitability for mass production [17]. Gold nanoparticles are widely utilized as nanoprobes due to their unique optical properties, easy synthesis and modification, and long-term storability [27,28]. By utilizing the wax printing method and AuNP, this proposed process was successfully applied to detect the AβO42 biomarker for the early monitoring of AD worldwide. The as-designed reaction chamber was of a circular shape with a 2.5 mm diameter, which is a hydrophilic area separated by wax hydrophobic patterns, acting as a centralized area for the dropping of sample solution. The copper-enhanced method on AuNP was confirmed by not only increasing the size of AuNP, but by changing the shape from spherical to polygonal [18,19]. Therefore, multi-chamber wax printed NCM was used as a sensor platform for running copper-enhanced gold immunoblotting, exhibiting dual signal amplification by the centralization ability of the reaction chamber, and colorimetric enhancement of the copper polygonal core–shell nanocomposite. The detection limit of this approach is up to 23.7 pg/mL with a smartphone camera and up to 320 pg/mL with the naked eye, which is comparable to recently developed nanobiosensors [29,30,31,32]. Additionally, there is no significant difference in the sensing calculation between the smartphone camera and the Chemidoc image system, suggesting that this colorimetric immunoblot could allow qualitative analysis by naked eye visualization, and quantitative analysis by cheaper devices, such as a smartphone camera, without the use of expensive equipment. This approach addresses the issue of global exploitation, owing to its advantages which include simplicity and potential for mass production, affordability, high availability, and freedom from the need for instruments or technical professionals.

## 5. Conclusions

The process from the fabrication of the paper-based sensing platform to the sensing application for AβO42 was successfully developed using the wax printing method and copper-enhanced Au nanoprobe immunoblotting. It is not only straightforward to fabricate the multi-chamber paper-based sensing platform by using the wax printing method but also timesaving and suitable for mass production. These designed multi-chamber platforms could concentrate the target sample in the desired size, to improve the sensing sensitivity. The copper-enhancing Au nanoprobe immunoblot was successfully developed for the highly sensitive detection of AβO42. This colorimetric blotting method based on a multi-chamber platform exhibited a strong capacity from the qualitative analysis with the naked eye to the quantitative analysis with the smartphone camera, suggesting promising potential in the sensing application for various biomarkers with good sensitivity, cost effectiveness, high availability, and independence from technical professionals and instruments. This developed process could serve as an applicable medical diagnostic tool worldwide for the accurate monitoring of AD, even in the poorest countries.

## Data Availability

The dataset generated and analyzed in this study is not publicly available, but may be obtained from the corresponding author upon reasonable request.
